# Anlotinib suppresses the DNA damage response by disrupting SETD1A and inducing p53-dependent apoptosis in Transformed Follicular Lymphoma

**DOI:** 10.7150/ijms.84952

**Published:** 2024-01-01

**Authors:** Xinguo Zhuang, Jingwei Yao, Xun Li, Yuelong Jiang, Mengya Zhong, Jinshui Tan, Hui Zhou, Genhong Li, Jie Zha, Bing Xu

**Affiliations:** 1 Department of Hematology, The First Affiliated Hospital of Xiamen University and Institute of Hematology, School of Medicine, Xiamen University, Xiamen, 361003, China.; 2 Key Laboratory of Xiamen for Diagnosis and Treatment of Hematological Malignancy, Xiamen, 361102, China.; 3 Department of Clinical Laboratory, The First Affiliated Hospital of Xiamen University, School of Medicine, Xiamen University, Xiamen, China.

**Keywords:** Transformed follicular lymphoma (tFL), Anlotinib, SETD1A, DNA damage response, p53

## Abstract

**Purpose**: The high tumor mutational burden (TMB) of transformed follicular lymphoma (tFL) leads to tumor heterogeneity and poor prognosis in follicular lymphoma, in which endogenous DNA damage and epigenetic modification are the key factors. This study aims to evaluate the efficacy of anlotinib in tFL and to investigate its potential therapeutic mechanism.

**Methods:** Cell viability and apoptosis were tested with CCK-8 and annexin V/PI staining kits, respectively. The tumorigenicity test in mice was utilized to further confirm the efficacy of anlotinib in vivo. Western blotting was utilized to explore the molecular mechanisms.

**Results:** Anlotinib induced G2/M phase arrest in tFL cells, inhibited the proliferation of tFL cells and promoted the apoptosis of tFL cells in a dose-dependent manner. Administration of anlotinib markedly reduced tumor mass and weight in an FL xenograft mouse model. The western blot and immunohistochemistry staining results confirmed that the mechanism by which anlotinib promoted tumor cell apoptosis was DNA damage. Further results showed that anlotinib significantly downregulated the expression of SETD1A, leading to its destruction. Anlotinib administration resulted in a significant dose-dependent increase in the level of p-p53. Furthermore, anlotinib greatly downregulated the antiapoptotic proteins Mcl-1 and in parallel upregulated the proapoptotic element BAX and Bak, accompanied by caspase-3 activation and PARP degradation.

**Conclusion:** Anlotinib has a good proapoptotic effect on tumor cells in vitro and in vivo, and its possible mechanism is related to the inhibition of the DNA damage response by disrupting SETD1A.

## Introduction

The most prevalent type of indolent nonlymphoma, Hodgkin's follicular lymphoma (FL), is defined by its slow growth and increased risk of high-grade transformation^1^-^3^. The histological transformation from FL to an aggressive lymphoma is expected to occur at a pace of 2 to 3% annually, finally, 20-25% of FL cases develop into high-grade B-cell lymphomas, namely transformed follicular lymphoma (tFL)^4^. The introduction of anti-CD20 monoclonal antibodies significantly improves the prognosis of FL^5^. However, 20% of patients develop exacerbations within 24 months of treatment, and half die within 5 years as a result of histological transformation^6^, ^7^. Patients with transformed follicular lymphoma (tFL), whose FL has evolved into a certain kind of clinically aggressive, high-grade B cell lymphoma such as DLBCL, Burkitt lymphoma, etc., suffered from poor therapeutic and clinical outcomes^4^, ^6^, ^8^, ^9^. It is recognized that patients undergoing transformation, especially those who are transforming to aggressive lymphoma, require different and potentially more aggressive targeted therapies. Unfortunately, patients diagnosed with tFL are often excluded from prospective clinical trials, which is why there is lack of information about the efficacy of new drugs for tFL^3^, ^5^, ^10^. Doctors do not know what the best treatment plan for tFL is, therefore, it is urgent to study how to treat tFL.

Although histological transformation (HT) refers to a biological event leading to high-grade, aggressive non-Hodgkin's lymphoma in patients with primary follicular lymphoma (FL)^11^, detailed genetic analysis has revealed that there is not a single mechanism driving the transformation of FL into tFL. Moreover, tFL is more likely to initiate from a series of transformations^10^, ^12^. The early driving factors of tFL are mainly epigenetic regulator mutation and t(14; 18) translocation, including MLL2, EZH2, and CREBBP^3^, ^10^, ^13^. Pasqualucci et al. revealed that the subsequent accumulation of oncogenic mutations may abet the “mutator” phenotype of FL. Subsequently, activation-induced deaminase (AID) was allowed to access inappropriate regions of the genome. It leads to discrete aberrant somatic hypermutation (aSHM), which is expressed as increased aberrant somatic hypermutation and aberrations in genes involved in cell cycle progression, proliferation and DNA damage response, such as TP53, MYC and CDKN2A in transformed samples^3^, ^10^, ^13^, ^14^. These data raise the possibility that epigenetic dysregulation may be the key to the development of HT. thus, targeting enzymes involved in the regulation of DNA methylation and histone modifications may be critical for developing more effective treatment strategies for tFL.

Anlotinib, as a novel oral multitargeting receptor tyrosine kinase inhibitor (TKI), mainly targets the intracellular ATP-binding site of VEGFRs (especially VEGFR2), FGFR1-4, PDGFR α/β, c-Kit, and Ret, preventing their phosphorylation and the subsequent activation of downstream signaling pathways to inhibit tumor angiogenesis and disease progression^15^. Interestingly, preclinical and clinical trials have shown that anlotinib exerts a broad spectrum of apoptosis-inducing effects in solid tumor cells, such as lung cancer cells, Intrahepatic cholangiocarcinoma (ICC) cells and hepatocellular carcinoma cells^16^-^18^. tFL, which has a similar morphology as these solid tumors, is a highly heterogeneous B-cell lymphoma. However, no studies have been conducted to estimate whether anlotinib exerts apoptosis-inducing effects in tFL. In addition, how anlotinib affects histone/chromatin modification, the cell cycle and the DNA damage response to induce cell apoptosis is currently unclear.

KMT2F (SETD1A) is present in a multiprotein complex (COMPASS) responsible for all H3K4 methylation^19^. The Polycomb and COMPASS families are well known for their opposing roles in balancing gene expression and are involved in several important biological processes, especially tumor pathogenesis^20^. SET domain containing 1A (SETD1A) is required for the appropriate expression of DNA damage response genes in AML cells^21^. According to previous studies, SETD1A is involved in DNA damage recognition and repair and maintains histone-H3-lysine-4 (H3K4) methylation on transcriptionally active promoters. Destruction of SETD1A expression results in a decrease in the expression of DNA damage response genes and induces apoptosis in a p53-dependent manner^21^. According to a genomic analysis of more than 100 types of cancer, FL was shown to have the eighth-highest tumor mutational burden and was highly correlated with endogenous sources of DNA damage^22^-^24^. Although anlotinib has shown good antitumor activity against multiple malignancy types in several preclinical and clinical trials, there is no research to evaluate its potential mechanism in the treatment of tFL. In this study, we are the first to explore the therapeutic effect of anlotinib on tFL and its possible mechanism. Our work provides promising evidence and a rationale for the clinical therapeutic application of anlotinib in the treatment of tFL.

## Materials and Methods

### Cell Lines and Molecules

Established human tFL cell lines, RL, DOHH2, SU-DHL4 and SU-DHL6, were purchased from Cobioer Biotechnology Company (Jiangsu, China) and cultured at 37 °C in a 5% CO_2_ incubator in RPMI-1640 medium (HyClone, Thermo Scientific, Logan, UT, USA) supplemented with 10% fetal bovine serum (FBS, Gibco, CA, USA). Anlotinib was obtained from Chia Tai Tianqing Pharmaceutical Group Co., Ltd., dissolved in DMSO (Invitrogen, Carlsbad, CA, USA) as a 10 mM stock solution for in vitro experiments and diluted in a 0.5% (w/v) CMC-Na suspension for oral gavage.

### Cell Viability Assessment

Cell Counting Kit-8 (CCK-8, APExBIO, Texas, USA) was used to assess cytotoxicity. In 96-well plates, 2×10^4^ cells/well were seeded in 100 µl of medium and treated with different concentrations of anlotinib for 24 h and 48 h. CCK-8 reagent (10 µl/well) was then added and incubated for another 2h before the absorbance at 450 nm was measured using a Bio-Rad microplate reader (Bio-Rad, CA, USA). The results of three separate triplicate experiments are presented as a percentage of viable cells compared to untreated controls. The IC50 values were calculated using GraphPad Prism 6 software.

### Analysis of the Cell Cycle and Apoptosis

Cells were treated with varying concentrations of anlotinib for the designated times. The cells were harvested and processed in accordance with the manufacturer's instructions. Propidium iodide (PI)/RNase staining buffer from BD Pharmingen (556463, New Jersey, USA) was used for cell cycle analysis. The cells were then examined using a NovoCyte flow cytometer with NovoExpress software (ACEA Biosciences, Inc., USA). An annexin V/PI apoptosis detection kit (BD Pharmingen, USA) was used to assess apoptosis. Cells positive for annexin V were determined to be apoptotic and were located in the dot plot's right quadrant. ANOVA was used for statistical analysis. P values less than 0.05 were considered significant when compared to the control group.

### Western Blot

After treatment with anlotinib for the designated times, the cells were lysed, and the protein concentration was determined using a BCA Protein Assay Kit (Pierce, Rockford, IL, USA) based on the methods previously reported^25^. Whole cell lysates (50 μg for each sample) were subjected to western blot analysis using the indicated primary antibodies and secondary HRP-conjugated antibodies (CA7074, 1:1000, Cell Signaling Technology, MA, USA). Blots were detected by visualization using the ECL Western Blotting Detection Kit (GeneFlow, Staffordshire, UK). The primary antibodies used were anti-β-actin (CA4970S, 1:1000, Cell Signaling Technology, MA, USA), anti-BAX (CA5023S, 1:1000, CST), anti-MCL1 (CA2538S, 1:1000, CST), anti-BAK (CA12105 1:1000, CST), anti-MYC (CA5605S 1:1000, CST), anti-PARP (CA9532, 1:1000, CST), anti-cleaved PARP (CA5625, 1:1000, CST), anti-caspase-3 (CA9662, 1:1000, CST), anti-cleaved caspase-3 (CA9661, 1:1000, CST), anti-p-p53 (Ser15) (CA9286, 1:1000, CST), anti-MDM2 (CA86934, 1:1000, CST), anti-γH2A.X (CA2577s, 1:1000, CST), and anti-SETD1A (ab70378, Abcam, Cambridge, UK) antibodies.

### In vivo Experiments

All animal procedures were carried out in accordance with the guidelines of Xiamen University's Animal Care and Use Committee and Ethics Committee. Female (nonfertile) BALB/C nude mice weighing 18-20 g were purchased from Xiamen University Laboratory Animal Center, Fujian, China. RL cells (150 ml of PBS, 2×10^6^ cells/mouse) were injected subcutaneously into the right flank of female BALB/C nude mice. The mice were separated into vehicle control and anlotinib groups randomly once the tumor volume reached 75 to 150 mm^3^ and then treated with either vehicle (PBS) or anlotinib (6 mg/kg/day) by oral gavage for 18 days. Every other day, the tumor size and body weight were measured. The formula used to determine tumor volumes was V=(L W^2^)/2 [V, volume (mm^3^); L, length (mm); and W, width (mm)].

After treatment, five mice from each group were sacrificed randomly, and tumor tissues were preserved and fixed with 4% paraformaldehyde (PFA) for hematoxylin-eosin staining (H&E staining). For immunohistochemistry (IHC) staining, paraffin-embedded slides were treated with Ki67 antibody (27309-1-AP, 1:2000, Proteintech, Suite, USA) overnight at 4 °C. DAB (DAB-2032, MXB Biotechnologies, Fujian, China) was utilized as directed for 5 min according to the manufacturer's instructions. TUNEL-FITC (A111-03, Vazyme Biotech, Jiangsu, China) was used for 20 min before examination using a fluorescence microscope (Nikon, Eclipse Ci-L, Japan). According to institutional guidelines, other mice were monitored until their tumors reached a size of 2000 mm^3^ or other humane endpoints (such as abscessed or necrotic tumors).

### Statistical Analysis

An unpaired t test was used to compare group pairs. One-way ANOVA was used to compare different groups. Each quantification was performed with at least three separate experiments. SPSS 25.0 and GraphPad Prism 6.0 were used for statistical analysis. The results with a p value less than 0.05 were statistically significant.

## Results

### Anlotinib induces G2/M arrest and inhibits proliferation of tFL cells

**Figure 1:**
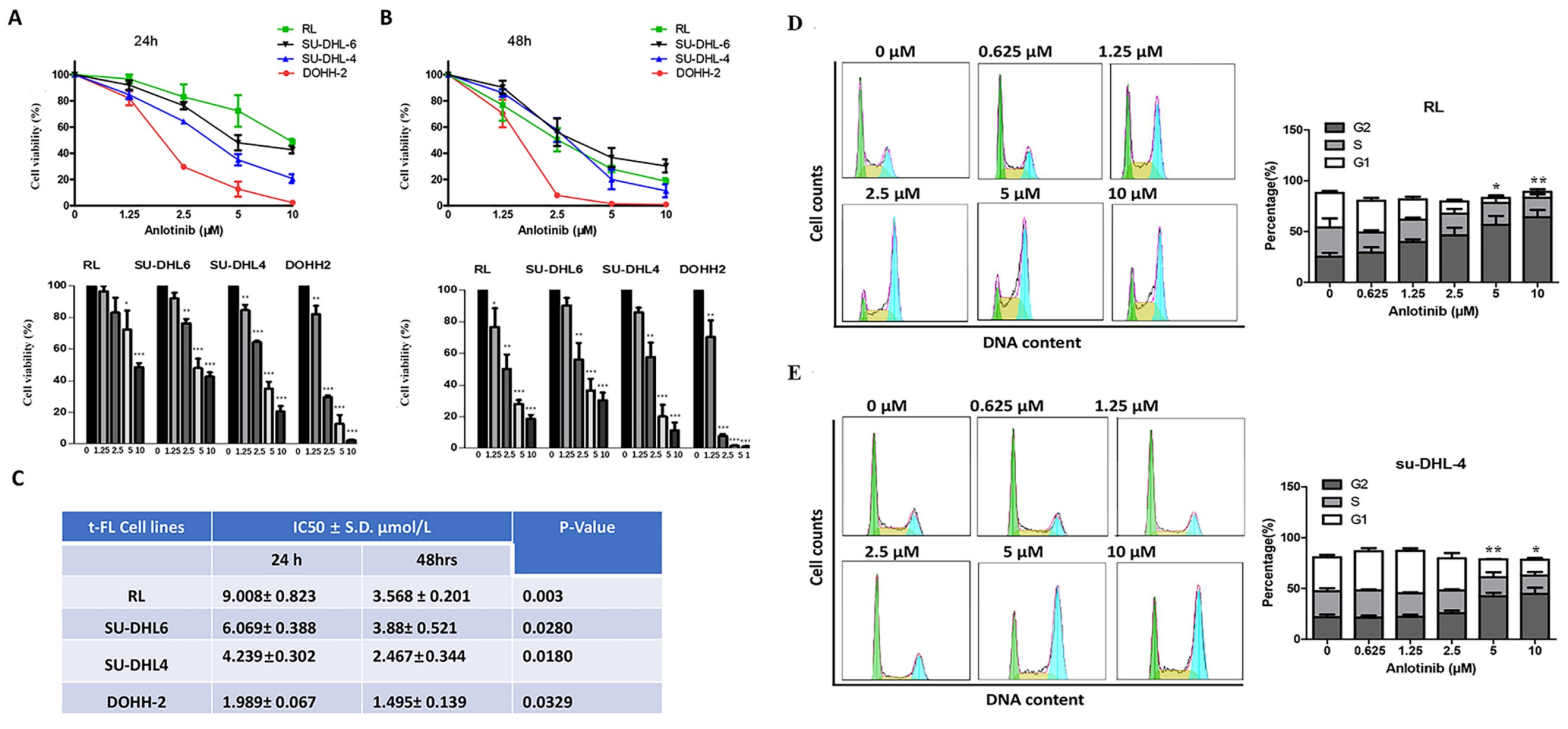
**Anlotinib inhibits the proliferative activity and induces G2/M arrest in tFL cells (RL, DOHH2, SU-DHL4 and SU-DHL6). A-B.** Anlotinib dramatically reduced the proliferative activity in tFL cell after 24 h and 48 h. **C.** The IC50 values of anlotinib in treated tFL cell lines. **D-E.** RL and SU-DHL4 cells were treated with the indicated concentrations of anlotinib for 18 h, and the cell cycle distribution in RL (D) and SU-DHL4 (E) cells was analyzed by flow cytometry. The right panel shows representative flow cytograms. (*P<0.05, **P<0.01, ***P<0.001, ****P<0.0001).

To probe the antitumor effect of anlotinib against FL, we first observed the cell viability effects of anlotinib on tFL cell lines (RL, DOHH-2, su-DHL-4 and su-DHL-6) using the CCK8 assay. Anlotinib significantly inhibited the proliferation of tFL cells in a dose-dependent fashion **(Fig. 1. A-B)**. The IC50 values of these cell lines ranged from 2 to 10 μM at 24 h and from 1.5 to 4 μM at 48 h **(Fig. 1. C)**, while the susceptibility to anlotinib was likely independent of the original FL and the subsequent aggressive FL subtypes, likely reflecting the heterogeneity of FL.

To further explore whether the inhibition of cell viability by anlotinib is related to cell cycle arrest, we next examined the effect of anlotinib on cell cycle arrest. We observed the cell cycle distribution of tFL cells (RL and su-DHL-4) after 18 hours of exposure to specified concentrations of anlotinib. The results revealed dose-dependent cell cycle arrest at the G2 checkpoint in RL and SU-DHL-4 cells **(Fig. 1. D-E)**. Taken together, these data suggested that anlotinib may be able to induce G2/M arrest and inhibit proliferation of tFL cells in a dose-dependent manner.

### Anlotinib promotes tFL cell apoptosis in vitro

**Figure 2:**
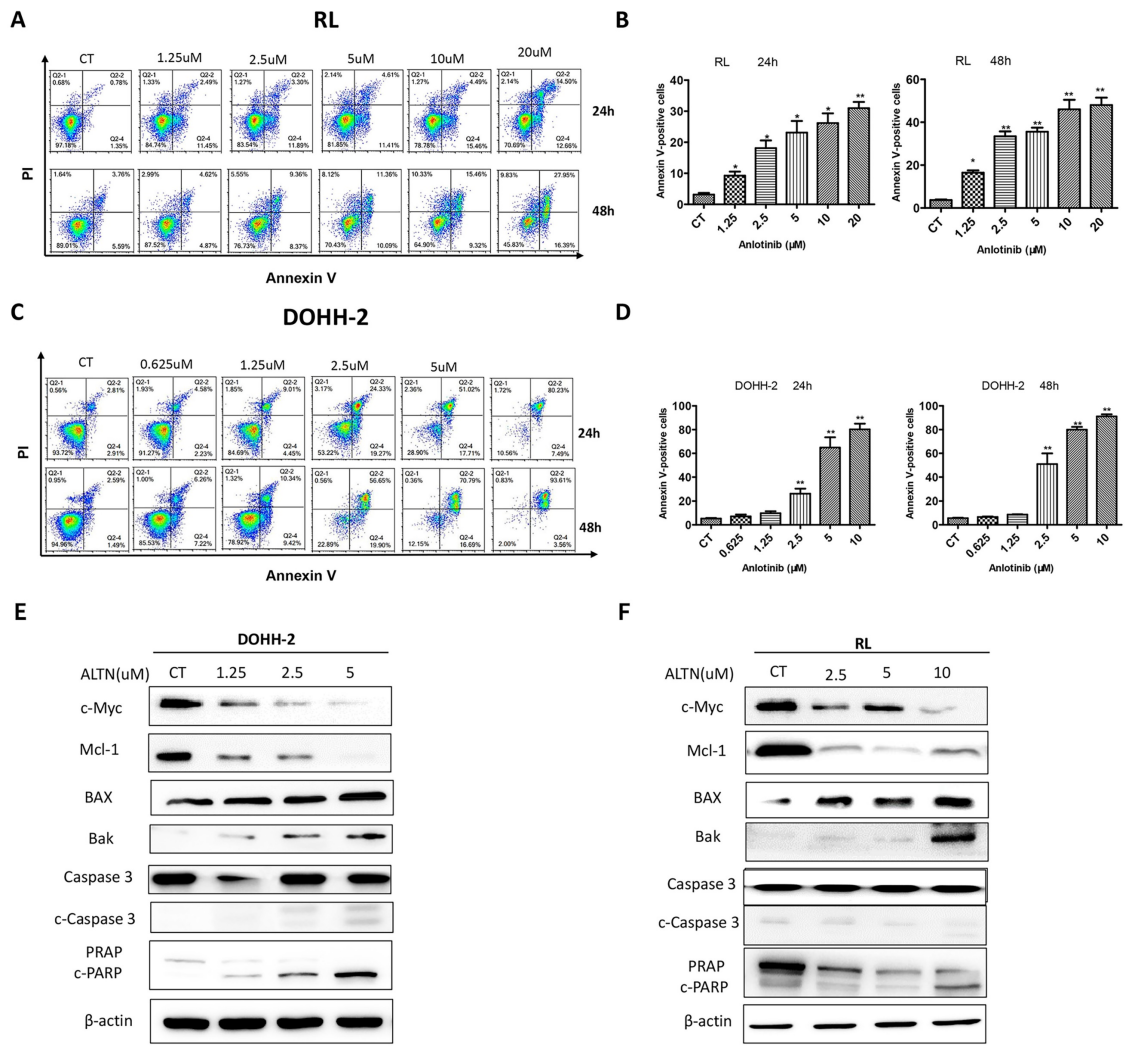
**Anlotinib promotes tFL cell apoptosis in vitro.** DOHH2** (A, B)** and RL** (C, D)** cells were treated with the indicated concentrations of anlotinib for 24 h or 48 h, and the percentages of apoptotic cells were then determined using annexin V/PI double staining. Apoptotic cells were significantly increased in DOHH2** (B)** and RL** (D)** cells after exposure to anlotinib at the indicated times. **E-F.** The expression of c-Myc, Mcl-1, BAX, Bak, PARP, cleaved PARP, caspase-3 and cleaved caspase-3 was assessed by Western blotting.

To further explore whether the cytotoxicity of anlotinib is related to the apoptosis of tFL cells, apoptosis was analyzed by flow cytometry with annexin V/PI double staining for 24 h and 48 h. In line with the results of cell cycle and viability, the results demonstrated that anlotinib largely increased the apoptotic percentage of tFL cells in a dose-dependent manner. Apoptosis was dramatically enhanced at 48 h in RL cells **(Fig. 2. A-B)**, however, no significant increase in apoptosis was detected in DOHH-2 cells at 48 h compared with 24 h after treatment **(Fig. 2. C-D)**. These data suggested that the induction of apoptosis might contribute to antitumor effect of anlotinib. In addition, we also analyzed the effect of anlotinib on the apoptotic signaling pathway, and the results showed that anlotinib greatly downregulated the antiapoptotic proteins Mcl-1 and in parallel upregulated the proapoptotic element BAX and Bak, accompanied by caspase-3 activation and PARP degradation **(Fig. 2. E-F)**.

### A regimen using anlotinib suppresses tumor growth in vivo in a xenograft model of tFL

**Figure 3:**
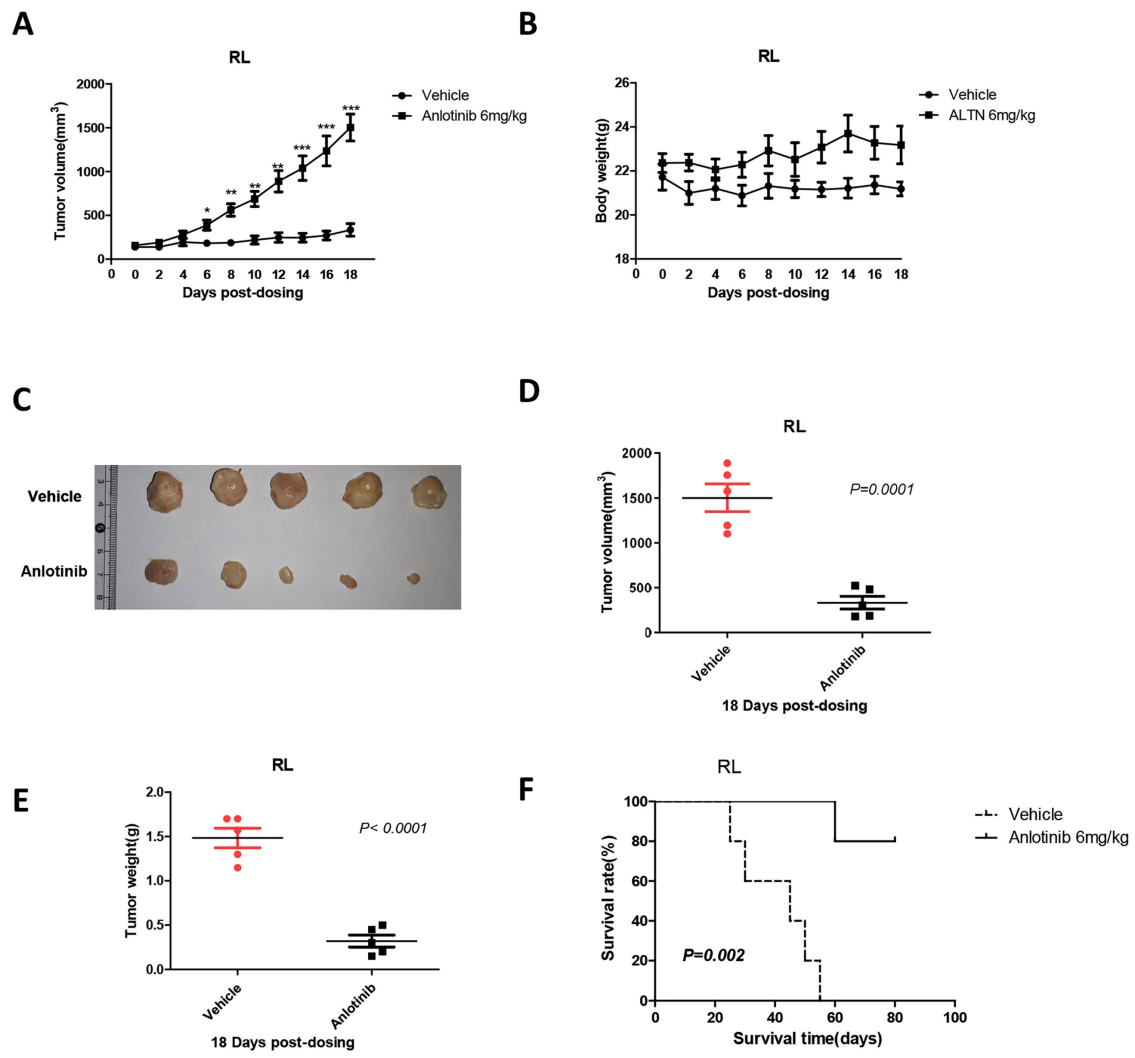
**Anlotinib suppresses tumor growth in vivo. A, C-E.** Anlotinib significantly reduced the tumor mass and weight in the tFL xenograft mouse model. **B.** Body weight changes of mice in the two groups before and after treatment. **F.** Anlotinib significantly prolonged the survival time of tFL xenograft mice.

To validate the ability of anlotinib to suppress tumor growth in vivo, we constructed a xenograft mouse model via subcutaneous inoculation with RL cells in BALB/C nude mice. When tumor volumes reached 100 mm^3^, the mice were randomly divided into two groups and treated with either vehicle or anlotinib. After 18 days of treatment with an oral dose of 6 mg/kg, it is noteworthy that compared with control mice, administration of anlotinib significantly reduced the tumor mass and weight in the tFL xenograft mouse model **(Fig. 3. A, C-E)** and significantly prolonged the survival time of mice **(Fig. 3. F)**. In addition, there was no remarkable weight distinction in mice during treatment between the anlotinib and control groups** (Fig. 3. B)**. Therefore, these results indicated that anlotinib inhibits tFL progression in vivo and is well tolerated.

### A regimen using anlotinib results in DNA damage in vitro and in vivo in tFL

**Figure 4:**
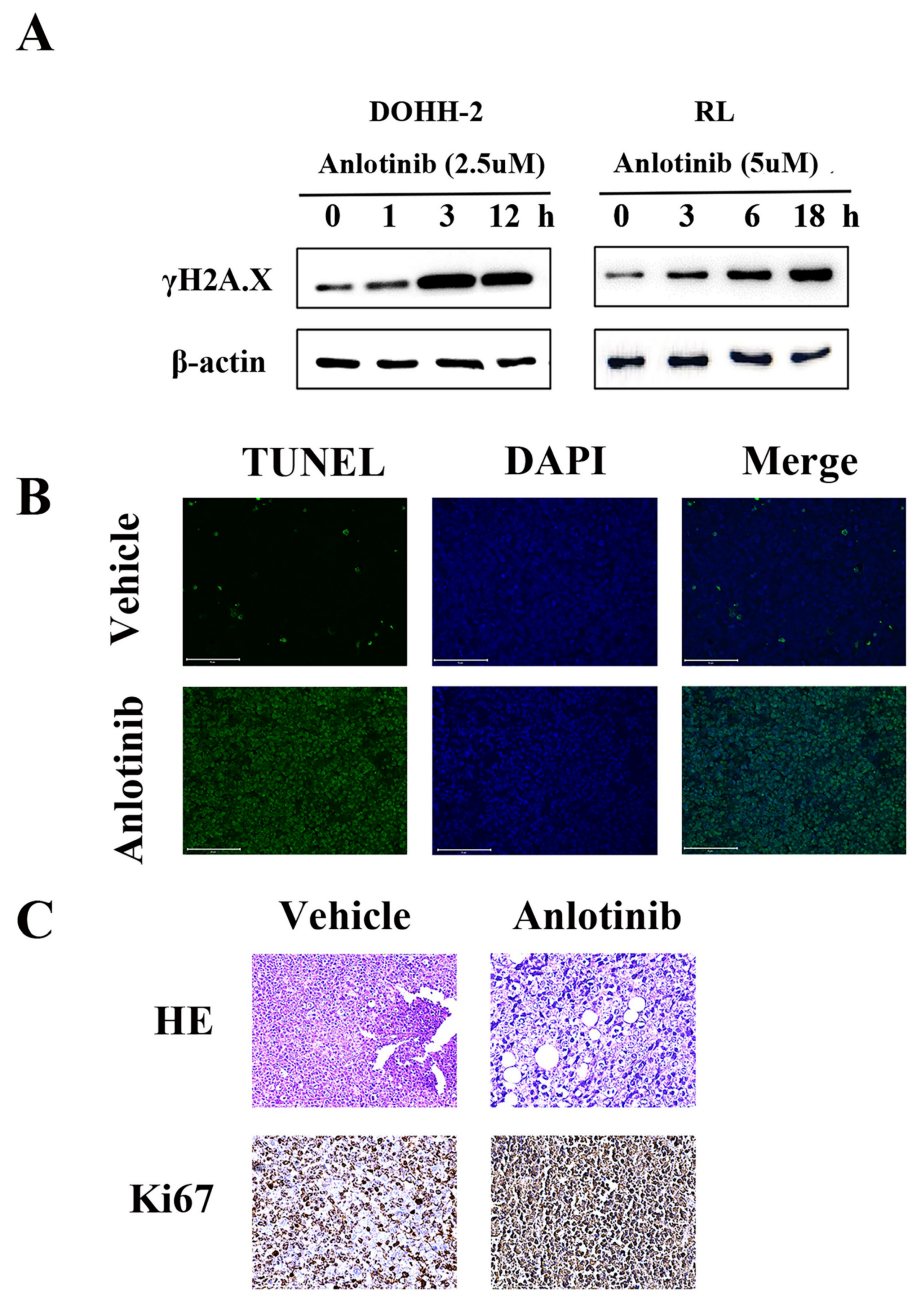
**Anlotinib results in DNA damage in vitro and in vivo in tFL. A.** Phosphorylation of γH2A.X was determined by Western blotting. **B.** Representative photos of the immunofluorescent TUNEL staining performed on serial tumor slices are displayed. **C.** Ki67 expression was determined using immunohistochemical staining; This is in comparison to H & E staining. Photos were taken using a Nikon microscope (original magnification, ×400).

DNA damage is a very important apoptosis-inducing factor. According to our previous research, the DNA damage response contributes to the anti-leukemia effect of anlotinib. To investigate the potential role of the DNA damage response (DDR) in the antitumor activity of anlotinib in tFL, western blot analysis was performed to monitor the expression of γH2A.X, a marker of DNA double-strand breaks** (Fig. 4. A)**. Phosphorylation of γH2A.X was significantly upregulated in the anlotinib treatment group. Consistent with the in vitro results, the tumor tissues obtained from the animals treated with anlotinib showed significant apoptosis in the form of DNA damage **(Fig. 4. B)**, as well as significant nuclear contraction, as determined by H&E staining. In addition, immunohistochemical staining of tumor tissues revealed that treatment with anlotinib strikingly decreased the expression of Ki-67, a cell proliferative index **(Fig. 4. C)**. Taken together, our results indicated that the regimen using anlotinib results in DNA damage in vitro and in vivo in tFL.

### Disruption of SETD1A suppresses the DNA damage response and induces p53-dependent apoptosis in tFL cells

**Figure 5:**
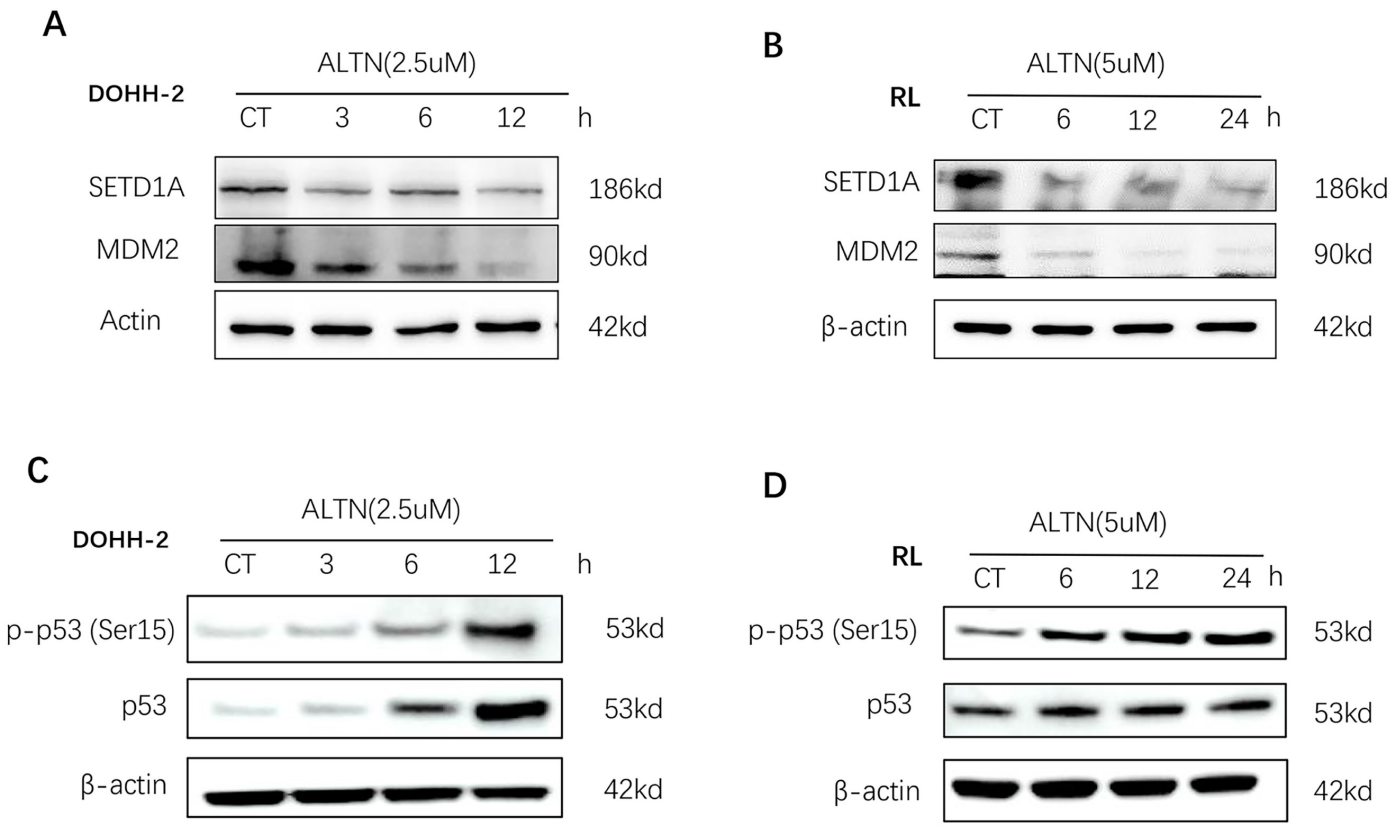
** Anlotinib (ALTN) suppresses SETD1A and induces the p53-dependent apoptosis pathway.** DOHH-2 and RL cells were incubated with 2.5 µM or 5 µM anlotinib for the indicated times, and the expression of SETD1A, MDM2 (**A-B**), p53 and phospho-p53 (Ser15) (**C-D**) were determined by western blotting.

Recent studies have shown that SETD1A is essential for maintaining DNA damage. Destruction of SETD1A expression inhibits the DNA damage response and induces p53-dependent apoptosis. To evaluate the role of SETD1A in the DNA damage of tFL cells induced by anlotinib, we first confirmed whether the expression of SETD1A is affected by anlotinib using Western blotting. As shown in **Figure 5 A-B**, anlotinib greatly downregulated the expression of SETD1A, leading to disruption of SETD1A. Since disruption of SETD1A could induce p53-dependent apoptosis, and we have proved that anlotinib promotes the apoptosis of tFL cells at the cellular and protein levels. Therefore, we also analyzed the effect of anlotinib on p53 expression. Our results confirmed that anlotinib administration resulted in a significant time-dependent increase in the level of p-p53 (Ser15) **(Figure 5. C-D)**. Overall, the antitumor effects of anlotinib might partially be attributed to impaired DNA damage responses through targeting SETD1A and p53.

## Discussion

Follicular lymphoma, with a high incidence in Western countries, is the second most common non-Hodgkin's lymphoma in the world^26^, ^27^. In the rituximab era, histologic transformation of follicular lymphoma remains the leading cause of follicular lymphoma-related mortality^28^. Patients always have a heterogeneous clinical course and poor response to treatment due to the random and multiple nature of their histological transition^4^, ^5^. Furthermore, in the absence of randomized clinical trials of tFL for many targeted agents, no optimal treatment for tFL has been confirmed. Therefore, we believe that the optimal treatment strategy for HT should not only target multiple important targets but also overcome heterogeneity. It has been reported that in preclinical studies, anlotinib has shown broad-spectrum antitumor effects in a variety of cancers^15^, ^29^, ^30^. In our study, we reported the feasibility of anlotinib in the treatment of tFL. The possible mechanism is that anlotinib inhibits the DNA damage response by disrupting SETD1A. Our research has greatly increased the possibility of developing tFL treatment and made it possible to improve the prognosis of tFL patients.

As a tyrosine kinase inhibitor, anlotinib inhibits tumor growth and angiogenesis by targeting VEGFR-2, VEGFR-3, and PDGFR-α^31^. Many studies have shown that anlotinib exerts a good antitumor effect in many cancers, such as osteosarcoma, thyroid cancer, and non-small cell lung cancer^16^, ^32^, ^33^. However, the antitumor effect of anlotinib in lymphoma, especially follicular lymphoma, is still unclear. The results of this research showed that anlotinib promoted the apoptosis of tFL cells in vivo and in vitro and inhibited tumor progression.

Since anlotinib inhibits the proliferation and induces the apoptosis of tFL cells, we further studied the extensive cytotoxic mechanism of anlotinib in tFL from the perspective of epigenetics to explore whether anlotinib regulates the cell cycle. Previous studies have shown that G2 arrest appears to be a common response of various tumor cells to anlotinib^17^, ^29^. In our research, we demonstrated that anlotinib induced a dose-dependent arrest of tFL cells in G2/M phase. In addition, c-Myc, a cell proliferation regulator that is rare in FL but appears more frequently in the process of transformation, was found to be downregulated after treatment with anlotinib. In general, the DNA damage response in cells is closely related to the cell cycle^34^, ^35^. In this study, γH2A, a molecular marker of DNA damage, was activated 1 hour before apoptosis in the DOHH2 cell line and 3 hours before apoptosis in the RL cell line. Consistent with the in vitro results, the results of TUNEL assay analysis revealed cellular DNA damage in the xenograft mouse model. Collectively, anlotinib can induce DNA damage in cells, which is likely related to the mechanism by which anlotinib inhibits cell proliferation, induces cell G2 arrest and promotes apoptosis.

SETD1A, a histone lysine methyltransferase, specifically methylates H3K4 and plays an important role in normal and cancer cell functions^36^. Many studies have shown that high expression of SETD1A is associated with poor prognosis in many cancer patients, but the mechanism of SETD1A related to the prognosis of cancer patients is different. Rui Wang and his colleagues found that SETD1A binds to the promoters of NEAT1 and EZH2 to activate gene transcription by inducing H3K4me3 enrichment, and the high expression of SETD1A represents a worse prognosis in patients with non-small cell lung cancer^37^. Another study on lung cancer showed that SETD1A was upregulated and was a key epigenetic modification factor for NSCLC cell proliferation, its deletion inhibited DNA replication^38^. In addition to its role in lung cancer, SETD1A also plays an important role in the progression of prostate cancer. SETD1A promotes the proliferation of mCRPC by regulating FOXM1 transcription^39^. There is considerable evidence to suggest that SETD1A may be a therapeutic target for many cancers^36^, ^40^.

The DNA damage response is a fundamental physiological mechanism aimed at protecting the genome of an organism itself, and most DDR pathways consist of several related and coordinated processes: detection of DNA damage, accumulation of DNA repair factors at the site of damage, and eventual physical repair. Compared with normal cells, the DDR has many key differences in cancer, and therefore many scientists believe that the DDR represents a good source of anticancer drug targets. In fact, similar drugs have emerged in recent years, such as the poly ADP ribose polymerase (PARP) inhibitor olaparib (Lynparza), which was approved for the treatment of BRCA1 or BRCA2 mutant tumors in 2015, the first drug based on this principle of medicine^36^, ^41^. In the case of DNA damage, previous studies have shown that the response of cells to DNA damage involves the cascading events of chromatin remodeling and histone modification, which coordinate repair^42^. Among them, SETD1A is thought to regulate p53 target genes to determine cell fate. p53 is known to be a determining transcription factor that selectively activates genes as part of a specific gene expression program to determine cellular outcomes^43^, ^44^.

However, in general, there are few studies on the SETD1A-mediated DNA damage response and tumor progression. Our previous studies have demonstrated that anlotinib impairs the DNA damage response by downregulating SETD1A in AML cells^29^. Similarly, in this study, we found that anlotinib has a similar response in tFL cells. Anlotinib greatly down regulated the expression of SETD1A, leading to disruption of SETD1A, accompanied by a significant time-dependent increase in the expression level of p-p53(Ser15). The overall trend in p53 expression levels was also up-regulated after anlotinib administration, which is compatible with previous studies^33^, ^45^, ^46^. Although the up-regulation trend of p53 expression is not as significant as p-p53, which may be due to different biological genetic backgrounds of different cell lines, given that p-p53(SER15) has a obvious pro-apoptotic effect on tumor cells^47^-^49^, these results have already revealed that anlotinib inhibited the DNA damage response and induced p53-dependent apoptosis in tFL cells by destroying SETD1A, suggesting the potential value of anlotinib in tFL.

In addition, our study confirmed that anlotinib can also regulate several recognized targets of the p53 pathway, including the downregulation of the antiapoptotic protein Mcl-1 and the upregulation of the proapoptotic proteins Bax and Bak in a manner similar to caspase-3 and PARP activation, thus inducing tFL cell apoptosis.

In conclusion, we explored the therapeutic effect of anlotinib on tFL and the possible mechanisms. We found that anlotinib has a good proapoptotic effect on tumor cells in vitro and in vivo, and its possible mechanism is related to the inhibition of the DNA damage response by disrupting SETD1A. Our study increases the possibility of developing tFL treatment and may improve the prognosis of tFL patients.
